# Opioid Prescription Claims Among Women of Reproductive Age — United States, 2008–2012

**Published:** 2015-01-23

**Authors:** Elizabeth C. Ailes, April L. Dawson, Jennifer N. Lind, Suzanne M. Gilboa, Meghan T. Frey, Cheryl S. Broussard, Margaret A. Honein

**Affiliations:** 1 Division of Birth Defects and Developmental Disabilities, National Center on Birth Defects and Developmental Disabilities, CDC

Prescription opioid use in the United States has become widespread (1), and studies of opioid exposure in pregnancy suggest increased risk for adverse pregnancy outcomes, including neonatal abstinence syndrome and birth defects (e.g., neural tube defects, gastroschisis, and congenital heart defects) (2,3). The development of birth defects often results from exposures during the first few weeks of pregnancy, which is a critical period for organ formation. Given that many pregnancies are not recognized until well after the first few weeks and half of all U.S. pregnancies are unplanned (4), all women who might become pregnant are at risk. Therefore, it is important to assess opioid medication use among all women of reproductive age. CDC used Truven Health’s MarketScan Commercial Claims and Encounters and Medicaid data* to estimate the number of opioid prescriptions dispensed by outpatient pharmacies to women aged 15–44 years. During 2008–2012, opioid prescription claims were consistently higher among Medicaid-enrolled women when compared with privately insured women (39.4% compared with 27.7%, p<0.001). The most frequently prescribed opioids among women in both groups were hydrocodone, codeine, and oxycodone. Efforts are needed to promote interventions to reduce opioid prescriptions among this population when safer alternative treatments are available.

CDC used Truven Health’s MarketScan Commercial Claims and Encounters and Medicaid data from 2008–2012 to assess outpatient pharmacy prescription drug claims for opioid-containing medications among reproductive-aged women (15–44 years). The Commercial Claims and Encounters data represent a convenience sample of employed persons with private employer–sponsored insurance and their dependents. The Medicaid data are an annual sample of Medicaid recipients in 10–13 states across the United States. Both data sources include person-level information (e.g., age, sex, and enrollment period) and claim-level data (e.g., outpatient pharmacy prescription claims). This analysis was restricted to women continuously enrolled (≥365 days) for the year under study in a private insurance or Medicaid plan that included prescription drug coverage. Outpatient pharmacy prescription medication claims were searched for opioid-containing medications using national drug codes; pure opioid antagonists (e.g., naloxone), medications that block the effects of opioids, were excluded when not combined with an opioid (e.g., buprenorphine/naloxone). For each woman, the prescription claims data were analyzed to determine whether she had ever filled a prescription for an opioid medication from an outpatient pharmacy during a given calendar year. The annual proportion of reproductive-aged women with outpatient prescription claims for an opioid during 2008–2012 was examined by health care coverage type and specific opioid medication, age group, U.S. geographic region (only available for privately insured women), and race/ethnicity (only available for Medicaid-enrolled women). The average proportion of reproductive-aged women who filled a prescription for an opioid from an outpatient pharmacy each year was also estimated. Chi-square tests were used to determine whether significant differences existed between the frequency of opioid claims among privately insured and Medicaid-enrolled women.

There were approximately 4.4–6.6 million privately insured and 0.4–0.8 million Medicaid-enrolled reproductive-aged women in the study sample each year during 2008–2012. Of these, on average 27.7% of privately insured and 39.4% of Medicaid-enrolled women filled a prescription for an opioid from an outpatient pharmacy each year (p<0.001). Opioid prescription claims were highest in 2009 with 29.1% of privately insured women and 41.4% of Medicaid-enrolled women filling a prescription for an opioid. During 2008–2012, opioid prescription claims were consistently higher among Medicaid-enrolled women when compared with privately insured women (Figure 1). In 2012, there were 0.7 and 1.6 prescriptions filled per woman among privately insured and Medicaid-enrolled women, respectively; of those who filled an opioid prescription, an average of 2.6 and 4.3 prescriptions were filled, respectively (Figure 2).

The most commonly prescribed opioids during 2008–2012 were hydrocodone (reported by an average of 17.5% of privately insured and 25.0% of Medicaid-enrolled women each year), codeine (6.9% and 9.4%), and oxycodone (5.5% and 13.0%) (Table). Privately insured women aged 30–34 years and Medicaid-enrolled women aged 40–44 years were most likely to fill prescriptions for opioids (Table). Among women with either type of health care coverage, women aged 15–19 years were least likely to fill a prescription for an opioid. Overall, for all age groups, women with Medicaid filled prescriptions for opioids more frequently than women with private insurance.

Significant regional and racial/ethnic differences were observed. Among privately insured women, opioid prescription claims were highest among those residing in the South. Among Medicaid-enrolled women, opioid prescription claims were highest among non-Hispanic whites (p<0.001) (Table).

## Discussion

Opioid-containing medications are widely prescribed among reproductive-aged women with either private insurance or Medicaid, with approximately one fourth of privately insured and over one third of Medicaid-enrolled women filling a prescription for an opioid each year during 2008–2012. In addition, CDC found an average of three opioids prescribed for every four privately insured women and nearly two opioid prescriptions for every one Medicaid-enrolled woman per year. This is a significant public health concern given evidence of adverse pregnancy outcomes with opioid exposure, the likelihood of exposures occurring among unrecognized or unintended pregnancies, and health care provider concerns about using other pain medications during early pregnancy (2,4,5).

This analysis presents data among all reproductive-aged women; however, previous studies from slightly earlier time periods have reported data among women who were actually pregnant. In general, more reproductive-aged women filled a prescription for an opioid in this study compared with what has been found among pregnant women. In a study of approximately 534,000 pregnant women with private insurance during 2005–2011, 14.4% filled a prescription for an opioid during pregnancy (6). In a study of approximately 1.1 million Medicaid-enrolled women nationwide with pregnancies during 2000–2007, 21.6% filled a prescription for an opioid from an outpatient pharmacy during pregnancy (7), and opioid dispensing increased over time. Similar to the findings in this report, these two previous studies of pregnant women also reported hydrocodone, codeine, and oxycodone as among the opioids most commonly dispensed by outpatient pharmacies.

The consistently higher frequency of opioid prescribing to Medicaid-enrolled women is of concern because approximately 50% of U.S. births occur to Medicaid-enrolled women (8). Other studies have found higher opioid prescribing rates to Medicaid-enrolled populations as compared with privately insured populations, but these have not specifically assessed use in reproductive-aged women. One study showed that, in 2005, 17% of a sample of persons aged ≥18 years enrolled in a multistate private insurance plan received an opioid prescription, compared with 30% of those enrolled in Arkansas Medicaid (9). These differences by health care coverage type might reflect differences in health plan drug formularies, differences in patient use of health care services based on health care coverage, or differences in the prevalence of underlying health conditions among Medicaid recipients compared with persons covered by employer-provided private health insurance.

Geographic region was available for the privately insured claims data and showed opioid prescription rates were highest among reproductive-aged women residing in the South and lowest in the Northeast. Race/ethnicity was available for Medicaid data, and indicated opioid prescriptions were nearly 1.5 times higher among non-Hispanic white reproductive-aged women than among non-Hispanic black or Hispanic women. Other reports of opioid prescribing patterns have shown similar geographic trends, with the South having the greatest number of prescription opioid claims (1,6,7), and white women being more likely than women of other racial/ethnic populations to fill an opioid prescription (7).

Although there appeared to be a decline in the frequency of opioids prescribed to both privately insured and Medicaid-enrolled women of reproductive age from 2009 to 2012, any conclusions about changes over time must be interpreted with caution. The apparent decline might indicate improvements in opioid prescribing practices; however, given the potential changes in the composition of the sample used for the privately insured claims data and in the states included in the Medicaid sample each year, this conclusion cannot be drawn from these data. At least one study has noted a decrease in opioid prescriptions among pregnant women, with a decline from 14.9% in 2005 to 12.9% in 2011 (6), although studies focused on earlier time periods have not noted such a decline (7,9). Notably, recently implemented federal regulation of certain opioid medications, such as hydrocodone, might decrease use in these populations (10).

The findings in this report are subject to at least four limitations. First, Truven Health’s MarketScan Commercial Claims and Encounters and Medicaid data are samples and might not be generalizable to the entire U.S. population. Although the privately insured claims data represent approximately 4–6 million insured persons, it is likely that they are only representative of persons with employer-based private insurance. The 10–13 states that contribute their data to the Medicaid sample each year do so anonymously. Therefore, changes over time in the Medicaid data might reflect changes in the sample of states, instead of broader changes among the Medicaid-enrolled population. It is also likely that certain women will be included in multiple years of either the Commercial Claims and Encounters or Medicaid data. Second, pregnant women were not identified in this analysis; the study population was based on female sex and age 15–44 years alone. Whether opioid prescriptions were limited to infertile or contracepting women was not ascertained. Third, these analyses likely underestimate opioid use, because the data only represent outpatient pharmacy claims. No information on inpatient opioid use, opioids obtained without a prescription, or opioids paid for out-of-pocket was available. Finally, although these data represent opioids dispensed by outpatient pharmacies, there was no verification that women actually took the medications.

What is already known on this topic?

Opioid use among women of reproductive age is a concern because opioid medications have been linked to birth defects and other adverse pregnancy outcomes. Given the high rate (approximately 50%) of unintended pregnancies in the United States, opioid use among reproductive-aged women can result in many early pregnancy exposures.

What is added by this report?

During 2008–2012, more than one fourth of privately insured and more than one third of Medicaid-enrolled reproductive-aged women (15–44 years) filled a prescription for an opioid from an outpatient pharmacy each year. Prescription rates were consistently higher among Medicaid-enrolled women when compared with privately insured women.

What are the implications for public health practice?

More targeted interventions and communications strategies are needed to reduce unnecessary prescribing and use of opioid-containing medications, particularly among women who might become pregnant.

This analysis used a large database to estimate the proportion of privately insured and Medicaid-enrolled reproductive-aged women who filled a prescription for an opioid from an outpatient pharmacy. Many women need to take opioid-containing medications to appropriately manage their health conditions; however, in some instances safer alternative treatments are available and use of opioids is unnecessary. Having a better understanding of prescription opioid use just before and during early pregnancy can help inform targeted interventions to reduce unnecessary prescribing of opioids and provide evidence-based information to health care providers and women about the risks of prenatal opioid exposure.

## Figures and Tables

**FIGURE 1:**
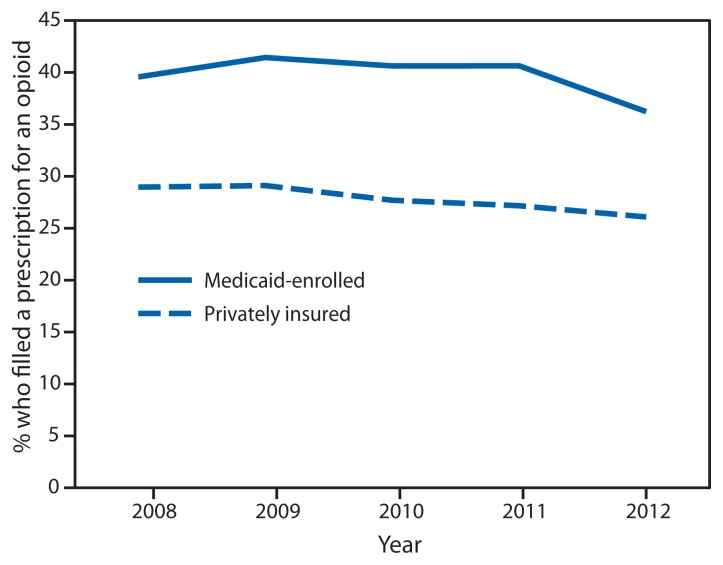
Percentage of women aged 15–44 years who filled a prescription for an opioid from an outpatient pharmacy, by health care coverage type and year — United States, 2008–2012 **Source:** Truven Health’s MarketScan Commercial Claims and Encounters and Medicaid data.

**FIGURE 2:**
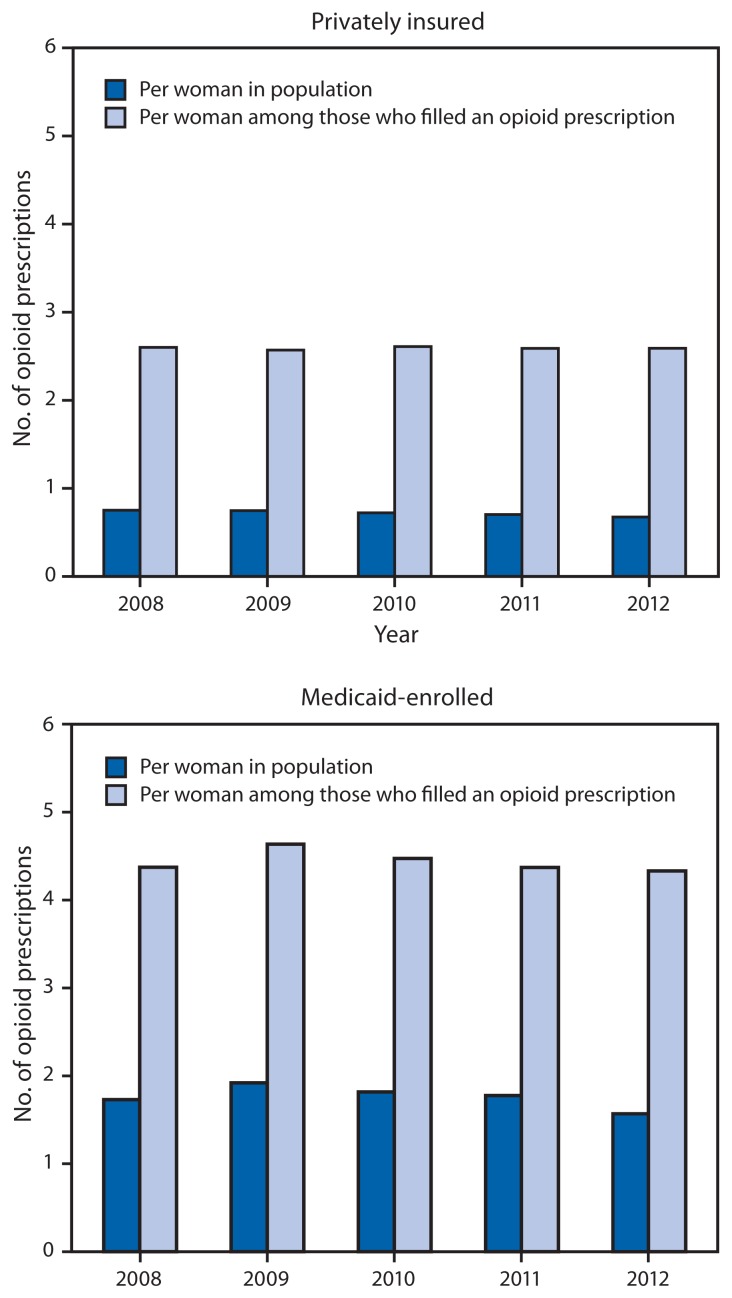
Average number of opioid prescriptions filled at an outpatient pharmacy per woman aged 15–44 years, among women with private insurance and Medicaid — United States, 2008–2012 **Source:** Truven Health’s MarketScan Commercial Claims and Encounters and Medicaid data.

**TABLE:** Percentage of women aged 15–44 years who filled a prescription for an opioid from an outpatient pharmacy, by health care coverage type and year — United States, 2008–2012

Characteristic	Privately insured						Medicaid-enrolled					
	
	2008	2009	2010	2011	2012	Average 2008–2012*	2008	2009	2010	2011	2012	Average 2008–2012*
**Total women** †	**4,440,181**	**5,225,282**	**5,635,375**	**6,417,512**	**6,598,518**		**422,602**	**552,425**	**568,802**	**534,976**	**803,920**	
**Age group (yrs)**												
15–19	21.2	22.0	20.2	20.1	19.3	**20.4**	25.3	26.9	26.8	26.1	24.6	**25.9**
20–24	25.8	26.2	23.9	23.1	22.0	**23.8**	41.6	44.5	43.5	43.0	37.8	**41.7**
25–29	31.2	31.1	29.7	29.1	27.6	**29.6**	46.5	50.0	48.6	48.3	41.9	**46.6**
30–34	32.1	31.9	30.8	30.7	29.7	**30.9**	47.8	51.8	50.5	50.6	44.9	**48.7**
35–39	31.4	31.4	30.3	30.0	29.2	**30.4**	50.0	53.1	52.1	53.4	46.4	**50.6**
40–44	31.0	30.8	29.9	29.6	28.8	**29.9**	52.5	54.4	53.6	56.4	48.0	**52.5**
**Geographic region** §												
Northeast	22.6	22.4	21.5	22.0	21.0	**21.8**						
North central	26.6	26.6	25.2	25.1	23.9	**25.4**						
South	32.2	32.7	31.8	30.7	30.4	**31.5**						
West	27.8	28.5	26.8	26.4	24.6	**26.6**						
Unknown	24.4	23.5	22.1	28.9	27.6	**27.3**						
**Race/Ethnicity** ¶												
White, non-Hispanic							49.2	49.7	48.1	45.7	42.1	**46.4**
Black, non-Hispanic							35.5	38.5	38.0	35.7	31.0	**35.2**
Hispanic							39.3	37.7	34.9	33.4	26.0	**33.6**
Other race							22.0	26.5	22.3	37.1	34.6	**28.0**
**Specific opioids** **												
Hydrocodone	18.0	17.8	17.3	17.6	16.9	**17.5**	22.9	25.1	26.4	27.0	23.9	**25.0**
Codeine	7.2	7.9	6.8	6.8	6.2	**6.9**	10.9	11.7	9.6	9.1	7.1	**9.4**
Oxycodone	5.2	5.4	5.6	5.7	5.5	**5.5**	13.2	13.4	12.7	13.4	12.5	**13.0**
Tramadol	2.4	2.5	2.7	3.3	3.4	**2.9**	6.8	7.9	8.5	9.7	8.9	**8.5**
Propoxyphene††	3.9	3.3	2.7	—	—	**1.8**	6.8	6.4	5.4	—	—	**3.3**
Hydromorphone	0.2	0.3	0.3	0.3	0.3	**0.3**	0.6	0.7	1.1	1.2	0.9	**0.9**
Meperidine	0.5	0.3	0.2	0.2	0.2	**0.3**	0.6	0.4	0.3	0.3	0.2	**0.3**
Morphine	0.2	0.1	0.1	0.1	0.1	**0.1**	0.8	0.6	0.6	0.5	0.5	**0.6**
Buprenorphine	0.1	0.1	0.1	0.2	0.2	**0.1**	0.2	0.3	0.3	0.4	0.5	**0.3**
Fentanyl	0.1	0.1	0.1	0.1	0.1	**0.1**	1.0	1.1	1.6	1.7	1.2	**1.3**
Tapentadol	0.0	0.0	0.1	0.2	0.2	**0.1**	0.0	0.0	0.1	0.0	0.1	**0.1**
Dihydrocodeine	0.1	0.1	0.1	0.1	0.0	**0.1**	0.1	0.1	0.1	0.0	0.0	**0.1**
Methadone	0.1	0.1	0.1	0.1	0.0	**0.1**	0.2	0.2	0.2	0.2	0.2	**0.2**

**Source:** Truven Health’s MarketScan Commercial Claims and Encounters and Medicaid data.

* The same woman might have been included in multiple years of data.

† Continuously enrolled (member days ≥365) in a plan that includes prescription drug coverage.

§ Geographic region is not included in Truven Health’s Medicaid data.

¶ Race/ethnicity is not included in Truven Health’s Commercial Claims and Encounters data.

** Not mutually exclusive; among prescriptions filled by at least 0.1% of privately insured or Medicaid-enrolled women on average each year during 2008–2012.

†† Discontinued after 2010.

## Footnotes

* Proprietary data on inpatient services and outpatient services and pharmacy claims provided by a convenience sample of commercial insurance providers and a sample of states (for Medicaid claims). Additional information is available at http://truvenhealth.com.
